# Memories for third-person experiences in immersive virtual reality

**DOI:** 10.1038/s41598-021-84047-6

**Published:** 2021-02-25

**Authors:** Heather Iriye, Peggy L. St. Jacques

**Affiliations:** 1 School of Psychology, University of Sussex, Brighton, UK; 2 Department of Psychology, University of Alberta, Edmonton, Canada; 3 Present Address: Department of Neuroscience, Karolinska Institutet, Solna, Sweden

**Keywords:** Psychology, Human behaviour

## Abstract

We typically experience the world from a first-person perspective (1PP) but can sometimes experience events from a third-person perspective (3PP) much as an observer might see us. Little is known about how visual perspective influences the formation of memories for events. We developed an immersive virtual reality paradigm to examine how visual perspective during encoding influences memories. Across two studies, participants explored immersive virtual environments from first-person and third-person avatar perspectives while wearing an Oculus Rift headset. Memory was tested immediately (Study One and Study Two) and following a one-week delay (Study Two). We assessed the accuracy of visual memory using cued recall questions and spatial memory by asking participants to draw maps of the layout of each environment (Study One and Study Two). Additional phenomenological ratings were included to assess visual perspective during remembering (Study Two). There were no differences in the accuracy of visual information across the two studies, but 3PP experiences were found to increase spatial memory accuracy due to their wider camera field of view when compared to 1PP experiences. Our results also demonstrate that 3PP experiences create 3PP memories, as reflected by an increase in subjective ratings of observer-like perspectives during remembering. In sum, visual perspective during memory formation influences the accuracy of spatial but not visual information, and the vantage point of memories during remembering.

## Introduction

We frequently experience and form mental representations of events from alternative points of view. For example, memories for events from our personal past can be retrieved from a first-person perspective (1PP), in which see the event as if through our own eyes, and from a third-person perspective (3PP) in which we see ourselves in the event as if from an observer’s point-of-view^1^. Previous studies have suggested that 3PPs during retrieval reflect changes that occur overtime in memories (e.g., a reduction in vividness), which make it difficult to reinstate the original 1PP from which memories are typically encoded. Supporting this idea, 3PPs are more frequent for remote than recent memories^2^. However, 3PP experiences can also naturally originate during the formation of memories that are highly negatively emotional or stressful, highly self-evaluative, and/or involve 3PP imagery during encoding^1,3^, and contribute to the increased frequency of 3PP memories in people with social phobia or post-traumatic stress disorder^4–6^. One challenge to empirical investigation of 3PP experiences and their impact on memory, is that by nature it is difficult to manipulate 3PPs during the formation of memories for events since people typically experience the world through their own eyes. Immersive virtual reality (VR) methodologies, which enable a sense of presence in a realistic environment with both a high level of ecological validity and experimental control^7^, provide a novel way to investigate the characteristics of 3PP experiences. Here, across two studies, we used immersive virtual reality to manipulate first-person and third-person avatar perspectives to investigate the influence of 3PP experiences on the formation of memories for events.

Previous research has shown that avatar perspective influences how virtual environments are experienced in two main ways that could impact memory. First, avatar perspective influences the sense of presence or the feeling of “being there” within a virtual environment, whereby one thinks, feels, and acts as though the virtual was reality^8^. Compared to 3PPs, 1PPs are associated with a higher sense of presence in virtual environments^9,10^. Lim and Reeves^11^ found that the relationship between the sense of presence and visual perspective depended upon whether participants were able to choose their virtual avatar or not. When participants were allowed to choose their avatar the sense of presence was higher in 3PPs than 1PPs, which could be due to the increased sense of ownership experienced when 3PP avatars become a proxy for the participant in the virtual environment. A stronger sense of presence during encoding may increase memory accuracy^12,13^. For example, Makowski and colleagues^13^ demonstrated that participants who reported a greater sense of presence while watching a movie also had better accuracy for factual details about the film (e.g., names of locations, actions, perceptual details, etc.), which they interpreted as attentional benefits on memory encoding when people are more absorbed in the to-be-remembered stimuli.

Second, avatar perspective affects spatial awareness in virtual environments^14–16^. For example, Gorisse and colleagues^14^ manipulated first-person and third-person avatar perspectives in a virtual environment and asked participants to deflect a series of projectiles and jump between platforms to activate terminals without falling. They found that third-person avatar perspectives led to faster response times, which they attributed to the improved spatial awareness of information in the periphery of the scene. One reason is that third-person avatar perspectives by definition involve a wider camera field of view (FOV) than first-person avatar perspectives, since the camera viewpoint is typically located some distance away from the position of the avatar in the virtual environment. Changes in spatial awareness due to avatar perspective could influence the types of details that people later recall, by increasing memory for peripheral information and/or the spatial layout of the overall scene.

Only a handful of studies have examined the impact of visual perspective during memory encoding^17–20^. In one study, Leynes and colleagues^19^ asked participants to study a list of words presented in a pre-recorded video in which they saw an empty chair in front of a desk with a computer screen displaying the words (1PP) or the same video with themselves sitting in the chair (3PP). In an immediate recognition memory test for the words, they found that 1PP experiences were associated with higher accuracy than 3PP experiences. In related research, Bréchet and colleagues^18^ demonstrated higher recognition memory accuracy following a 1-h delay in memories for 1PP experiences for events formed in a virtual environment that included the participant’s body (i.e., seeing their arms) compared to 1PP experiences that did not include the body, but a 3PP condition was not included (also see^20^). Bergouignan, Nyberg, and Ehrsson^17^ demonstrated that 1PPs for realistic social interactions contributed to subjective feelings of recollection during retrieval. The authors investigated 1PP and 3PP experiences for events in which participants sat in front of an actor playing an eccentric professor who they verbally engaged with in four oral examination style interviews. They manipulated visual perspective in real-time by asking participants to wear a VR headset that was fed by a camera located behind and slightly above the participant’s head to mimic what the participant would be able to see from their own eyes without the headset (1PP) or located in front of the participant such that both the participant’s physical body and actor could be viewed during the event (3PP). The authors manipulated perceived self-location by repeatedly moving a rod just below the camera and simultaneously touching the participant’s chest prior to the oral interviews. One week later, participants were asked to freely recall the events and then to rate the subjective sense of recollection on separate categories of information. They found that memory for 3PP experiences was associated with an overall reduction in the subjective sense of recollection of the events when compared to memories for 1PP experiences, which was driven by reductions in emotional, spatial, and temporal information about the events. In a separate fMRI study, Bergouignan et al.^17^ further showed that reductions in the subjective characteristics of memories for 3PP experiences were associated with changes in the response of the hippocampus during retrieval. In sum, only two studies have directly examined the influence of first-person versus third-person perspectives on memory formation, and these studies suggest that memories for 1PP experiences are associated with more accurate recognition memory and a greater subjective sense of recollection, particularly following a delay.

While the handful of previous studies provide a preliminary understanding of how visual perspective during encoding influences memories for events they also raises several key questions about the nature of memories for 3PP experiences. One question is whether the retention interval differentially affects the influence of 3PP experiences on memory formation. While Leynes et al.^19^ reported differences due to visual perspective during memory encoding on an immediate memory test, Bergouinan et al.^17^ found differences only following a one-week delay. Another important question is how 3PP experiences influence the accuracy of different types of information recalled during memory retrieval. Visual perspective affects the features that are visible in events with potential consequences on memory^21^. For example, compared to 1PPs, 3PPs include greater information about the physical body and its location in the wider context of the scene due to the zoomed out viewpoint on the event. Adopting a 3PP during memory is associated with changes in the nature of spatial information, with greater recall of information about the spatial relationships between objects^22^, but reduced accuracy of the spatial relationship of objects in relation to the self^23^. Additionally, Libby and Eibach^24^ proposed that 1PP imagery is associated with a greater focus on concrete features, whereas 3PP imagery is associated with broader contextual information. Visual information is also reduced when adopting a 3PP compared to 1PP during retrieval^25,26^. For example, Marcotti and St. Jacques^25^ showed that reductions in vividness ratings associated with adopting a 3PP during memory retrieval contributed to less accurate memory on a subsequent cued-recall test. In these studies, however, 3PPs reflect a manipulation of visual perspective during retrieval or the natural adoption of a 3PP in memories for 1PP experiences^27^. Although Bergouinan et al. did report reductions in subjective vividness in memories for 3PP experiences it remains unknown whether these reductions reflect changes in the objective accuracy of visual information and whether 3PP experiences also influence spatial aspects of memory.

Finally, an important unanswered question is whether 3PP experiences lead to the creation of 3PP memories. A number of researchers have argued that 3PP memories might be formed during memory encoding^1,3^. Although previous studies have shown that it possible to manipulate visual perspective during memory encoding, none to our knowledge have demonstrated that visual perspective during encoding is preserved when memories are later remembered. For example, Bergouignan et al.^17^ did not find significant differences in the visual perspective of memories formed during 1PP compared to 3PP experiences. That is, participants did not report stronger 3PPs when remembering events experienced from a 3PP. One reason may be that visual perspective was measured on a single rating scale that treated first-person and third-person perspectives as dichotomous constructs, which might not have captured the complexity of potential changes in viewpoint due to differences in visual perspective during memory encoding. In fact, a number of researchers have argued that 1PP and 3PP are independent variables^28^ that should be measured using separate scales^29^.

The main aims of the current research were to investigate how 3PP experiences affect the accuracy of visual and spatial information (Study One and Study Two), the influence of the retention interval on memory for 3PP experiences (Study Two), and the vantage point associated with memories for 3PP experiences (Study Two). These novel questions were investigated across two studies that examined the influence of avatar perspective during the formation of memories in virtual environments. Participants experienced immersive virtual environments from a first-person or third-person perspective of an avatar while wearing a virtual reality headset (i.e., Oculus Rift), and rated the sense of presence they felt in each environment. Memory was tested immediately following the exploration of the virtual environments (Study One & Study Two) and after a week-delay (Study Two). In both studies, we assessed memory in two main ways: 1) using a map-drawing task of the environment to assess spatial memory, and 2) using a cued-recall test to assess memory for visual details. Based on evidence suggesting that third-person avatar perspectives change the focus of attention to information in the periphery^14^ and related work suggesting that adopting a 3PP during imagery leads people to think in more concrete ways^24^, we also manipulated whether cued-recall questions examined central (e.g., identification of a main object in the scene) or peripheral (e.g., weather outside the window in the scene) information. Across both studies, we predicted that the subjective sense of presence would be higher in virtual environments experienced from a 1PP compared to a 3PP, which was expected to lead to an increase in visual cued-recall memory accuracy for memories for 1PP versus 3PP experiences^13^. Additionally, we also predicted that experiencing virtual environments from different viewpoints would influence the type of information that people retrieved in memories—leading to lower accuracy for the spatial layout of the scene in memories for 1PP than 3PP experiences^14,22^, and higher accuracy for central than peripheral visual details in memories for 1PP than 3PP experiences^24^.

In Study One, we additionally manipulated avatar choice to explore its interactive effect on 1PP and 3PP experiences. We predicted that allowing participants to choose their avatar would lead to higher presence when forming memories of virtual environments from a 3PP compared to 1PP^14^, and that this increased sense of presence would attenuate differences in memory accuracy between the perspective conditions^13^. In Study Two, we used bespoke avatars that physically resembled the participants and manipulated the retention interval to test the effects of delay on accuracy and phenomenology of memories for 1PP and 3PP experiences, as well as including additional subjective ratings to assess visual perspective during memory retrieval. We predicted that differences in memories due to visual perspective during memory encoding would be stronger following a delay, given research suggesting that 3PP experiences lead to a less durable memory representations that emerge over time^17^. Additionally, we predicted that 3PP experiences would increase the tendency to adopt a 3PP during remembering, as reflected by a pattern of higher 3PP and lower 1PP ratings of visual perspective.

## Methods: study one

### Participants

Participants included 50 healthy young adults with no prior history of neurological or psychiatric impairment, and who were not taking medications that affected mood or cognitive functioning. One participant experienced virtual reality sickness and withdrew from the study. Thus, the final sample was 49 participants (32 women, mean age in years = 21.80, SD = 2.59). All experimental protocols were approved by the Sciences & Technology Cross-Schools Research Ethics Committee (SCITEC C-REC) at the University of Sussex. All methods were carried out in accordance with these guidelines, and informed consent was obtained from all participants.

### Materials

**Figure 1:**
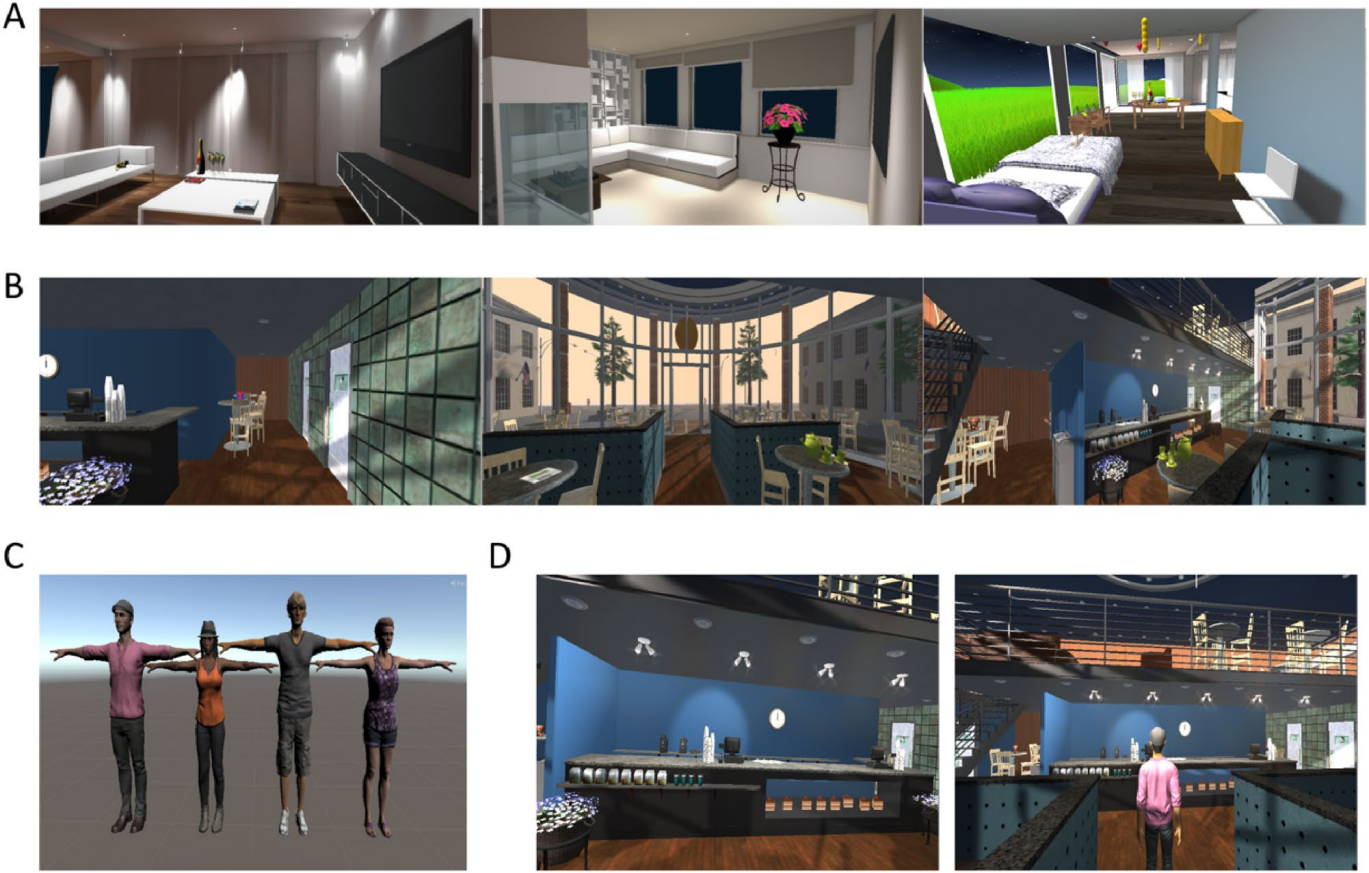
Study one virtual environments and avatars. (**A**) Screenshots from the virtual house environment. (**B**) Screenshots from the virtual café environment. (**C**) Four avatars that participants could choose from. (**D**) Screenshots of the first-person (left) and third-person (right) avatar perspectives.

A virtual house and café (see Fig. 1A,B) were downloaded from the Unity Asset Store. Visual details were then added and modified in terms of size and color using Unity Engine 5.1.3. For the café, we added cakes to the display case, travel mugs on the counter, a clock on the wall behind the counter, a garbage can by the bathrooms, as well as a tea set, teddy bear, handbag, newspaper, and present placed on various tables throughout the scene. On the street outside the café, we included a statue of a horse visible from the right café windows and adjusted the sky to resemble a sunset/sunrise. For the house environment, we created a birthday party scene by adding a large table in the living room that included a cake, plates, champagne, flutes, and party horns. The living room was further furnished with a wooden chair, desk with party hats, television and couch. Balloons of two different shapes and colors floating against the living room ceiling were also visible in the scene. We created a bedroom to the house that included a bed with a floral duvet, a bureau with flowers on top of it, and a chair. There was a library next to the bedroom, where a chess board and flower stand were inserted. Outside the house, we created a forest with a large, grassy hill in the distance. Lastly, the sky was changed to a starry night sky. To control for potential differences in complexity between the two environments, we counterbalanced the perspective they were viewed from across participants. Stimuli were viewed through an Oculus Rift DK2 head mounted display (HMD). This HMD has a resolution of 960 × 1080 per eye displayed at 75 Hz with 100° field of view. A package of four virtual avatars (see Fig. 1C) was downloaded from the Unity Asset Store. Avatars were modified by skin tone, hair color, and gender for each participant.

### Procedure

Participants were allocated to one of two groups. In the No Choice group, participants were assigned an avatar that matched their gender to use throughout the experiment based on their gender (*N* = 24). In the Choice Group, participants were instructed to choose the avatar they felt most resembled them from the four selections, which were customized to match each participant’s skin tone and hair color (*N* = 25). We manipulated whether or not participants were able to choose their avatar because previous research has demonstrated that it increases physiological responses within virtual environments^11^, which may affect the level of immersion.

During the study phase, participants sat in front of a desktop computer while wearing the HMD to view the immersive VR environment. Participants used keyboard presses to move the avatar’s body while the head tracking sensors in the HMD mapped head movements in real time. Visual perspective was manipulated using a within-subjects design by changing the camera location in the VR environment. Participants viewed the environments either from a (1) 1PP, as if from the viewpoint of the avatar’s eyes, or (2) 3PP, located five meters behind the avatar (see Fig. 1D). We used an incidental memory task, wherein participants were guided by the experimenter around the virtual environment (e.g., enter the café, stop just inside the door, look around, etc.; for full scripts see Supplemental Material S1) and asked to search for a red key they were told was inside the scene. There was no red key in either environment. Participants used keyboard presses to move the avatar’s body while the head tracking sensors in the HMD mapped head movements in real time. Each environment was explored for approximately three minutes and all participants were able to follow the experimenter’s instructions and complete the virtual tour.

Immediately after exploring each virtual environment, participants were asked several questions related to the degree of presence they felt within the virtual environment^30^. There were three questions designed to target core subjective components of presence: (1) the sense of “being there” (from 1 = not at all, to 7 = very much so), (2) the number of times that the virtual environment became reality, such that the real world was almost forgotten (from 1 = none, to 7 = many), and 3) the sense that the virtual scene was a location visited as opposed to images on a screen (from 1 = images, 4 = mixed, to 7 = locations)^8^. We calculated a mean presence rating separately for each condition by averaging responses to individual questions. After answering the questions about presence, participants had a two-minute break before entering the next virtual environment. The order of virtual environments and the perspective they were viewed from was counterbalanced across participants.

After exploring both virtual environments and making online ratings of the sense of presence for these experiences, participants were then tested on their memory for each environment. First, participants were asked to write a narrative description of their memory for each virtual environment (i.e., to describe their memory for the virtual environments in as much detail as possible). Second, spatial memory accuracy was assessed by asking participants to draw the spatial layout of each environment from a survey (i.e. bird’s eye) perspective, as if they were looking down on the environment from above on a map. Finally, visual memory accuracy was assessed using cued-recall questions that pertained to either central or peripheral details of the virtual environment, presented in random order. Central details were defined as those aspects of the environment that directly related to the search task (i.e., questions about color, number, and identify of objects placed where a key was likely to be located). Peripheral details referred to aspects of the virtual environment that were not related to the search task, such as the weather, time of day on the clocks, and color of the walls. Participants were asked 14 questions for each environment, with an equal number of questions referring to central and peripheral details. Participants were also asked to rate how confident they were in their to answers each question on a scale from 1 (low) to 5 (high). We calculated average confidence ratings for central and peripheral details separately.

### Data analysis

Spatial maps drawn by participants were coded based on a master spatial map that included the correct label and position of the test environments’ features (i.e., furniture, doors, and walls). One point was awarded for each correctly labelled feature in the correct position (10 total per environment), and the percentage of correct responses was calculated for each participant. Interrater reliability was assessed by calculating an intraclass correlation coefficient based on spatial memory accuracy scores obtained by two independent raters on half of the data, randomly selected. The intraclass correlation coefficient was 0.95, indicating a high degree of interrater reliability. Responses to the cued recall questions were also coded for accuracy. We used a strict criterion in which responses had to exactly match the correct response in order to be scored as accurate (e.g., *What beverage was being served?* Correct Answer: *Champagne*, Incorrect Answer: *Wine*). The percentage of correct responses for central and peripheral details for both perspective conditions was calculated for each participant. We also conducted an exploratory analysis to investigate whether avatar perspective during memory formation influenced the language used in narrative recall (see Supplementary Information S1).

## Results: study one

### Sense of presence

**Figure 2:**
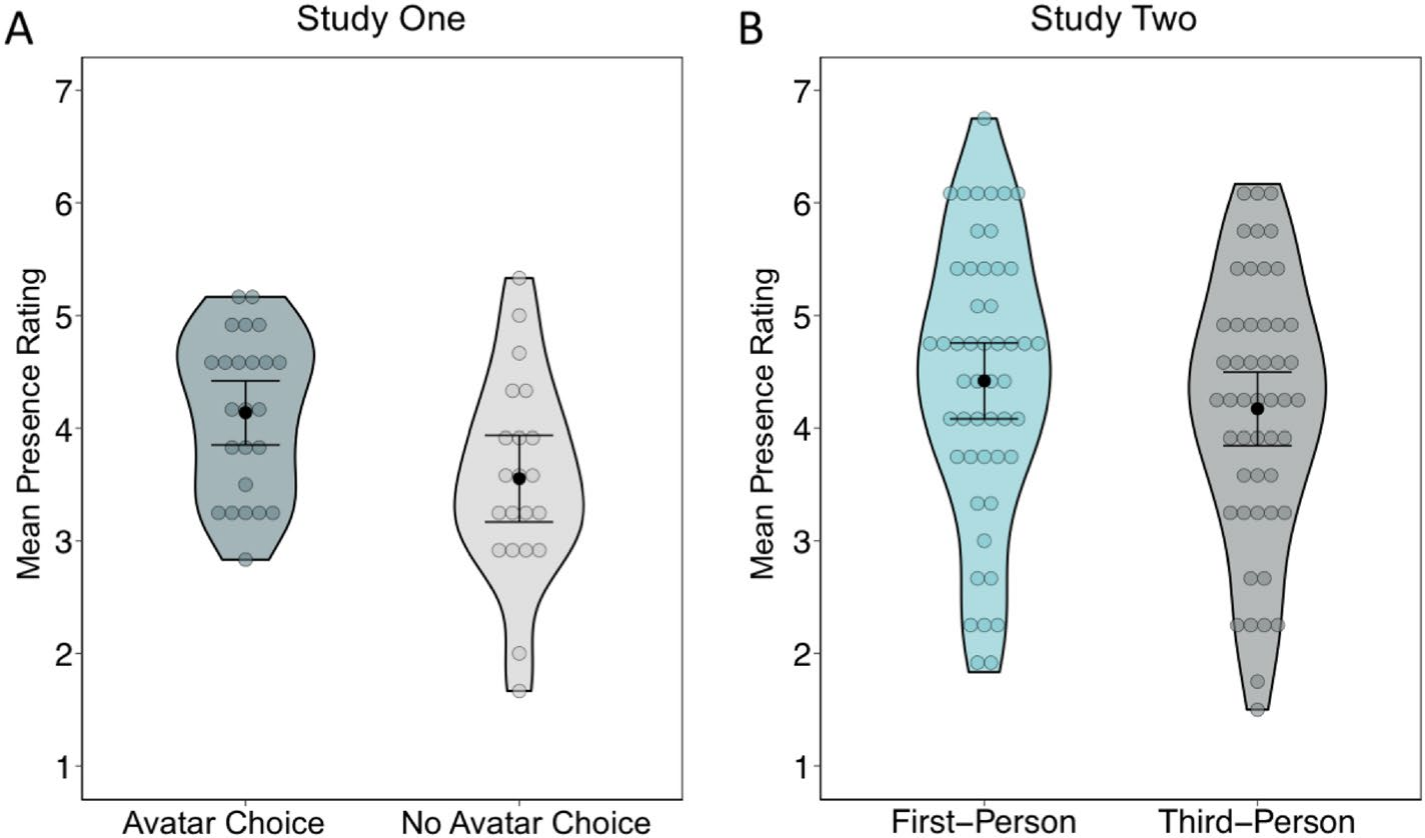
Sense of presence. (**A**) In Study One, there was a significant increase in the sense of presence when participants were able to choose their avatar compared to no choice. (**B**) In study Two, there was a significant increase in the sense of presence when virtual environments were experienced from a first-person compared to a third-person avatar perspective. Colored circles reflect mean for each participant, black circles represent the mean within each condition, and error bars reflect the 95% CI.

We conducted a 2 (Perspective Condition: 1PP, 3PP) × 2 (Avatar Group: Choice, No Choice) mixed repeated measures ANOVA with avatar choice as a between-subjects factor and visual perspective as a within-subjects factor on the average presence ratings (for means and SD see Table 1; presence ratings were not collected in 4 participants; data available at: https://doi.org/10.17632/8wkpyxb7th.1). There was a main effect of avatar choice, *F* (1,43) = 4.93, *p* = 0.032, $\eta_{p}^{2}$ = 0.10, which reflected a higher sense of presence in the choice (*M* = 4.11, *S.D* = 0.71) than the no choice group (*M* = 3.57, *S.D* = 0.92; see Fig. 2A). There were no other main effects or interactions.

**Table 1:** Average sense of presence ratings.

	Study 1				Study 2	
	Avatar choice		No avatar choice		Mean	SD
	Mean	SD	Mean	SD		
First-person	4.28	1.12	3.87	1.08	4.42	1.22
Third-person	3.93	1.11	3.27	1.34	4.17	1.17

### Memory accuracy

**Figure 3:**
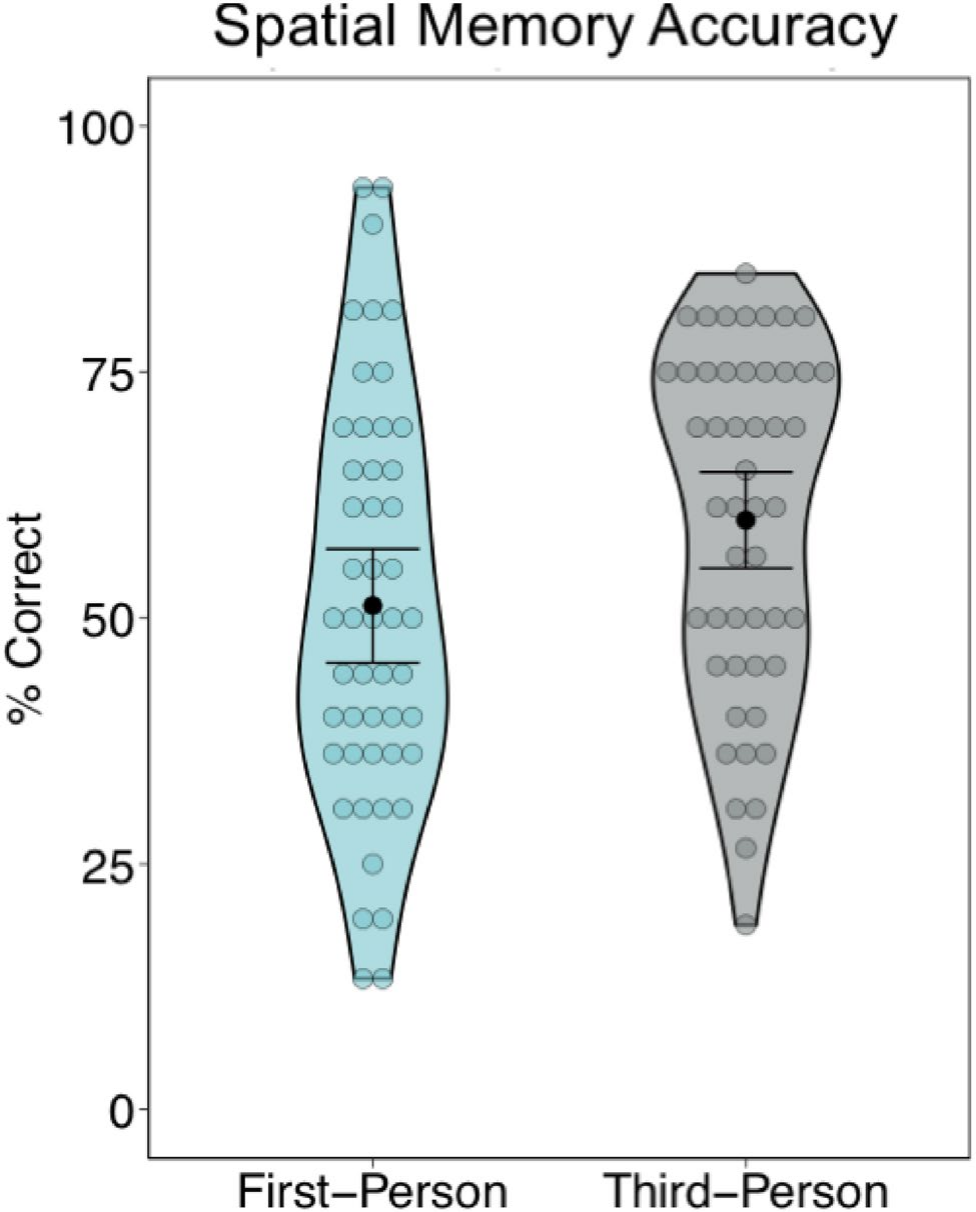
Spatial Memory Accuracy. There was a significant increase in spatial memory accuracy in the third-person compared to the first-person avatar perspective condition. Colored circles reflect mean for each participant, black circles represent the mean within each condition, and error bars reflect the 95% CI.

To examine the influence of visual perspective on spatial memory accuracy, we conducted a 2 (Perspective Condition: 1PP, 3PP) × 2 (Avatar Group: Choice, No Choice) mixed repeated measures ANOVA with avatar choice as a between-subjects factor and visual perspective as a within-subjects factor (see Table 2 for means and SDs). There was a significant main effect of perspective condition, *F* (1,47) = 6.17, *p* = 0.02, $\eta_{p}^{2}$ = 0.12, indicating higher spatial memory accuracy for events experienced from 3PPs (*M* = 59.94, *SD* = 17.44) relative to 1PPs (*M* = 51.26, *SD* = 20.60; see Fig. 3). There were no other main effects of interactions.

**Table 2:** Spatial memory accuracy (% correct).

	Study 1				Study 2			
	Avatar choice		No avatar choice		Immediate		Delayed	
	Mean	SD	Mean	SD	Mean	SD	Mean	SD
First-person	49.71	22.25	52.86	19.06	39.50	19.27	39.20	18.94
Third-person	57.68	17.63	62.29	17.30	43.40	19.02	37.60	16.23

To examine cued-recall accuracy, we conducted a 2 (Perspective Condition: 1PP, 3PP) × 2 (Detail: Central, Peripheral) × 2 (Avatar Choice: Choice, No Choice) mixed ANOVA with avatar choice as a between-subjects factor and visual perspective and detail as within-subjects factors separately on the percentage of correct responses and confidence ratings (see Table 3 for means and SDs). There were no significant effects.

**Table 3:** Cued-recall memory accuracy and confidence ratings.

	Study 1				Study 2			
	Avatar choice		No avatar choice		Immediate		Delayed	
	Mean	SD	Mean	SD	Mean	SD	Mean	SD
**Accuracy (% correct)**								
First-person								
Central	47.44	15.79	47.71	22.87	40.00	25.56	33.20	21.99
Peripheral	48.64	22.86	44.63	23.82	28.00	17.61	26.80	20.84
Third-person								
Central	45.20	18.06	54.17	20.78	44.40	18.20	32.80	23.82
Peripheral	42.64	18.60	45.83	23.00	30.40	20.70	26.40	21.17
**Confidence ratings**								
First-person								
Central	2.71	0.50	2.79	0.70	3.18	1.16	2.50	0.96
Peripheral	2.77	0.67	2.83	0.88	3.34	1.22	2.82	1.21
Third-person								
Central	2.66	0.69	3.09	0.70	3.38	1.05	2.43	1.02
Peripheral	2.80	0.63	2.77	0.83	3.25	1.20	2.83	1.09

## Discussion: study one

The results of Study One demonstrate that avatar choice influenced the subjective sense of presence in the virtual environment. Participants who were able to choose their avatar reported a stronger sense of presence in the virtual environment, perhaps reflecting a greater sense of identification of their virtual avatar compared to participants who were assigned an avatar. Inconsistent with previous research^9^, the sense of presence was not significantly higher when people experienced the virtual environment from a 1PP rather than a 3PP, nor was there an interaction between avatar choice and visual perspective as some studies have found^11^. One reason may be that participants did not feel like their avatars physically resembled them^14^, even if they were allowed to choose one, which meant that self-identification with the avatar was not strong enough to produce effects of visual perspective on presence within the virtual environments. To increase the sense of self-identification, in Study Two we created bespoke avatars to match each participant’s appearance and confirmed the effectiveness of this procedure by asking participants to rate how much they identified with their avatar. To further boost self-identification with the avatar, we employed real-time motion capture implemented with an Xbox Kinect camera that allowed participants to stand and naturally move their virtual avatar within the virtual environments. Finally, we also included a training period in which participants viewed their virtual doppelgänger in a mirror from 1PPs and 3PPs prior to experiencing the virtual environment from that same viewpoint.

**Figure 4:**
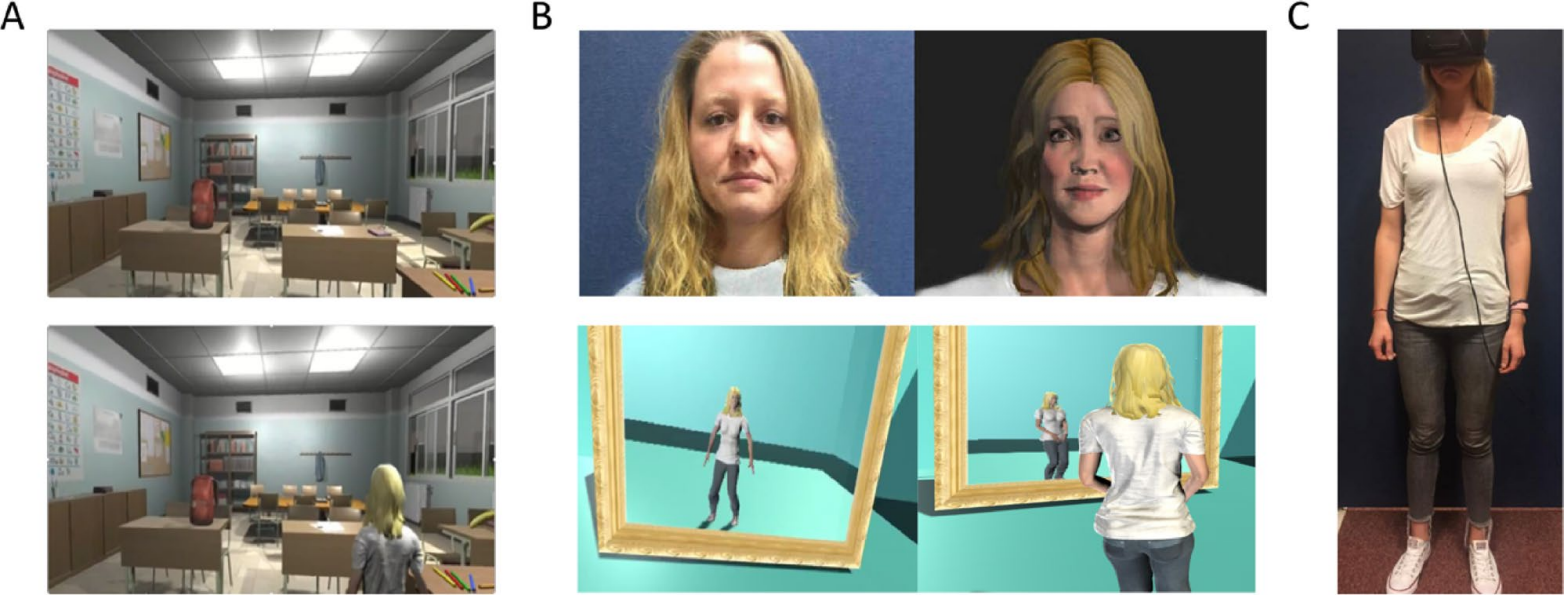
Study two virtual environment and avatars. (**A**) Examples of the first-person (above) and third-person (below) avatar perspectives when the camera field-of-view is controlled for. (**B**) Example of bespoke avatars created to physically resemble the participant (above) and mirror training environment from first-person (below left) and third-person (below right) avatar perspectives. (**C**) During the task, participants wore a white t-shirt and jeans to match the clothing worn by their bespoke virtual avatar, and a Kinect for Xbox was used to move the virtual avatar using their physical body movements. Written informed consent for open access publication was obtained for using this identifying image.

We found that spatial memory accuracy for the layout of the virtual environments was higher when virtual events were experienced from a 3PP compared to a 1PP. By nature, 3PPs involve a wider camera FOV (see Fig. 1D), which may influence how the surrounding spatial aspects of the virtual environment are experienced. Supporting this finding, Gorisse and colleagues^14^ demonstrated that experiencing a virtual environment from a third-person perspective located above and behind a virtual avatar, as in the current study, led to improved spatial awareness due to the heightened ability to perceive objects in the periphery of the scene enabled by a wider camera FOV. Here, the wider camera FOV in the 3PP condition also allowed participants to perceive more of the virtual scene, potentially contributing to more accurate retrieval of the spatial layout. To directly test whether the increase in spatial memory accuracy was dependent on the wider camera FOV in the 3PP condition, in Study Two we artificially manipulated the camera FOV so that it was equivalent in the two perspective conditions (compare Fig. 4A with Fig. 1D).

There were no significant differences between 1PPs and 3PPs on visual memory accuracy based on cued recall. Adopting a 3PP during memory retrieval has been shown to decrease the subjective sense of vividness, which is thought to reflect a loss of visual information overtime in memories originally encoded from a 1PP^27^. Bergouignan and colleagues^17^ also showed that memories encoded from a 3PP reduced the subjective sense of recollection and vividness when tested after a delay. Together these findings suggest that adopting a 3PP may lead to less durable memories, such that differences in visual perspective during encoding might only emerge following a delay. Additionally, visual perspective during memory encoding might affect subjective rather than objective aspects of visual information. To address these possibilities, in Study Two we included an additional retention interval to test memory after a one-week delay, along with the inclusion of subjective ratings (i.e., visual perspective, emotional intensity, vividness, and reliving), in addition to the objective measures used in Study One, to investigate the influence of visual perspective on the phenomenology of memory retrieval.

## Methods: study two

### Participants

Participants included 54 healthy young adults with no prior history of neurological or psychiatric impairment and who were not taking medications that affected mood or cognitive functioning. Four participants were excluded due to incorrect counterbalancing, which meant that these individuals did not receive the full number of experimental conditions. Thus, the analysis was conducted on 50 participants (32 women, mean age in years = 22.71, SD = 3.37). All experimental protocols were approved by the Sciences & Technology Cross-Schools Research Ethics Committee (SCITEC C-REC) at the University of Sussex. All methods were carried out in accordance with these guidelines, and informed consent was obtained from all participants.

### Materials

Personalized avatars were built from recent full-body photographs supplied by participants in advance of the experiment using Adobe Fuse CC (see Fig. 4B; written informed consent for open access publication was obtained for using this identifying image), uploaded to Mixamo in the Adobe Creative Cloud, and imported into Unity Engine 5.2.2. During the testing session, each participant’s movement was captured using Brekel Probody V1 linked to an XBox Kinect camera positioned 1.2 m in front of the participant (see Fig. 4C; written informed consent for open access publication was obtained for using this identifying image). The location of the participant’s joints was tracked in real time and projected onto the participant’s avatar in the virtual environment. A training environment containing a virtual mirror was created so that participants could familiarize themselves with the VR environment and increase the sense of self-identification with their avatar before entering the test environments (see Fig. 4B).

Virtual test environments included the café and house from Study One. We included two additional virtual environments to test the effects of delay on memory: a classroom and an office, which were downloaded from the Unity Asset Store. Modifications to the virtual classroom included adding a set of colored pencils, a backpack, banana, textbooks, notebooks, paper, an eraser, backpack, and globe on top of various desks. Student desks behind the first row were deleted and replaced by a large table surrounded by chairs. An alphabet and periodic table poster were added to the walls. A swing set was placed in the courtyard outside the classroom visible from the windows and the sky was set to an overcast day. Modifications to the office included placing reading glasses, house plants, and water bottles on desks in the scene, a clock and world map on the walls, a laptop on a bench located near the window, a water cooler next to one end of the bench, and a large plant at the other end of the bench. Skyscrapers and a sunny sky with some white clouds were visible outside the window. To control for potential differences in complexity between virtual environments, we counterbalanced which condition they were associated with across participants. Stimuli were viewed through an Oculus Rift DK2 HMD with the same settings as Study One.

### Procedure

The study involved two separate sessions spaced one-week apart. During session one, participants were asked to wear a plain white t-shirt and blue jeans to the laboratory to match their avatars’ outfits (see Fig. 4B,C). A t-shirt was provided for participants that did not have one. Participants wore the HMD and were initially placed on a marked position in the lab 1.2 m in front of the Xbox Kinect motion capture sensor. Participants were then immersed inside a virtual training room whereby they could see the full body of their avatar from either a 1PP or 3PP in a mirror (see Fig. 4C). The 3PP was positioned five meters behind the avatar at the same height as the 1PP. Camera FOV was held constant across both perspectives by moving the start position of the avatar in the 1PP condition to the location of the camera in the 3PP condition. Participants were then guided through a script specifying a series of movements by the experimenter for 45 s (i.e., lifting up each arm and leg, looking down with their head, stepping towards the mirror and looking at their avatar in the mirror, crouching down and standing, jumping up, and taking two steps back from the mirror). Following training, participants were asked to close their eyes while the experimenter closed the mirror environment and opened the test environment, which took no longer than five seconds. The participant was then cued to open their eyes and visually search the virtual environment for a red key for a duration of two minutes. Participants were instructed to make head movements, but to remain on their mark (i.e., instructed not to walk) in order to control for the amount of movement between participants and minimize potential VR sickness. Participants were prompted to continue actively searching the virtual environment in the event they were not making head movements. After the two minutes of visual search were complete, participants rated the sense of presence they experienced in the virtual environment. This process was repeated until the participant had experienced each of the four virtual environments. Immediately after exploring each virtual environment, participants completed the same set of questions assessing presence as in Study One. Thus, two of the virtual environments were experienced from a 1PP and two from a 3PP. The order in which the virtual environments were presented and the perspective from which each was viewed was counterbalanced. Participants were given a two-minute break between virtual environments.

After exploring all of the virtual environments, memory for half of the virtual environments was tested immediately, whereas the remaining half of the virtual environments were tested following a delay of one week. To control for potential effects of rehearsal, unique virtual environments were tested at each delay. Thus, two virtual environments (one 1PP and one 3PP) were tested immediately, whereas the remaining two virtual environments (one 1PP and one 3PP) was tested after a delay. Memory was tested similarly to Study One except that in Study Two participants were also asked to provide subjective ratings of visual perspective (separately for own eyes and observer perspectives), vividness (i.e., the clarity with which participants could see the event in their mind), reliving (i.e., the degree to which participants could feel or experience the environment again as if it were happening right now, or as if they were mentally traveling back in time to when the event occurred), and emotional intensity (i.e., the strength of emotions, regardless of how positive or negative) on seven-point Likert scales following their narrative account. For visual perspective ratings, participants were instructed that an own eyes perspective reflected “seeing it from the viewpoint of my virtual avatar,” whereas observer perspectives reflected “seeing my virtual avatar in the environment.”

At the end of session one, participants also answered two questions about how strongly they self-identified with their virtual avatar on seven-point Likert scales (i.e., “to what extent do you think your avatar actually resembles you?”; “to what extent did you identify with your avatar, as in you felt that you were the avatar in the virtual environment?”).

### Data analysis

Data was analyzed identically to Study One (data available at: https://doi.org/10.17632/8wkpyxb7th.1). Participant responses from the two avatar identification questions were averaged together to obtain an overall avatar identification rating. There was a moderately high level of self-identification with the virtual avatar (*M* = 4.52, SD = 1.29), indicating that the creation of personalized avatars was effective for creating a sense of identification with the avatar.

## Results: study two

### Sense of presence

We conducted a paired t-test to investigate potential differences in the effects of visual perspective on the sense of presence in the virtual environments. The sense of presence was higher in the 1PP compared to the 3PP condition, t (49) = 2.18, p = 0.034, effect size = 0.31 (see Table 1 and Fig. 2).

### Memory accuracy

To examine the influence of visual perspective on spatial memory accuracy, we conducted a 2 (Perspective Condition: 1PP, 3PP) × 2 (Test: Immediate, Delayed) repeated measures ANOVA (see Table 3 for means and SDs). There were no significant effects.

To investigate cued recall performance, we conducted a 2 (Perspective Condition: 1PP, 3PP) × 2 (Test: Immediate, Delayed) × 2 (Detail: Central, Peripheral) repeated measures ANOVA separately on the percentage of correct responses and confidence ratings (see Table 3 for means and SDs). For correct responses, there was a main effect of detail, *F* (1,49) = 25.56, *p* < 0.001, $\eta_{p}^{2}$ = 0.34, reflecting higher accuracy for central (*M* = 37.60, *SD* = 10.31) compared to peripheral (*M* = 27.90, *SD* = 12.08) details. There was also a main effect of time, *F* (1,49) = 8.21, *p* = 0.006, $\eta_{p}^{2}$ = 0.14, reflecting higher accuracy when memory was tested immediately (*M* = 35.70, *SD* = 10.97) relative to a one-week delay (*M* = 29.80, *SD* = 12.08). Turning to confidence ratings, there was a main effect of detail, *F* (1,49) = 5.52, *p* = 0.02, $\eta_{p}^{2}$ = 0.10, reflecting lower confidence ratings for central (*M* = 2.87, *SD* = 0.71) compared to peripheral (*M* = 3.06, *SD* = 0.90) details. There was also a main effect of time, *F* (1,49) = 42.94, *p* < 0.001, $\eta_{p}^{2}$ = 0.47, reflecting higher confidence ratings when memory was tested immediately (*M* = 3.29, *SD* = 0.87) relative to a one-week delay (*M* = 2.64, *SD* = 0.80). The main effects were qualified by a detail x time interaction, *F* (1,49) = 4.65, *p* = 0.04, $\eta_{p}^{2}$ = 0.09. Bonferroni corrected post-hoc analysis showed that confidence ratings were higher for peripheral compared to central details after a delay, *p* = 004, but there was no difference when memory was tested immediately, *p* = 0.94. There were no other main effects or interactions for cued recall accuracy or confidence.

### Subjective ratings

One motivation for study 2 was to investigate how events perceptually experienced from 1PPs and 3PPs influences visual perspective during later memory retrieval. We examined the average visual perspective ratings during memory retrieval in a 2 (Perspective Rating: Own Eyes, Observer) × 2 (Perspective Condition: 1PP, 3PP) × 2 (Test: Immediate, Delayed) repeated measures ANOVA (for means and SD see Table 4).

**Table 4:** Subjective memory ratings.

	Immdediate		Delayed	
	Mean	SD	Mean	SD
**Own eyes**				
First-person	5.14	1.74	5.46	1.40
Third-person	3.84	2.04	4.46	1.89
**Observer**				
First-person	2.50	1.83	2.08	1.28
Third-person	4.30	2.12	3.24	1.89
**Vividness**				
First-person	4.24	1.56	3.50	1.40
Third-person	4.00	1.41	3.46	1.37
**Reliving**				
First-person	4.20	1.74	4.10	1.51
Third-person	4.00	1.32	4.06	1.39
**Emotional intensity**				
First-person	3.00	1.28	2.94	1.46
Third-person	3.08	1.69	3.04	1.54

**Figure 5:**
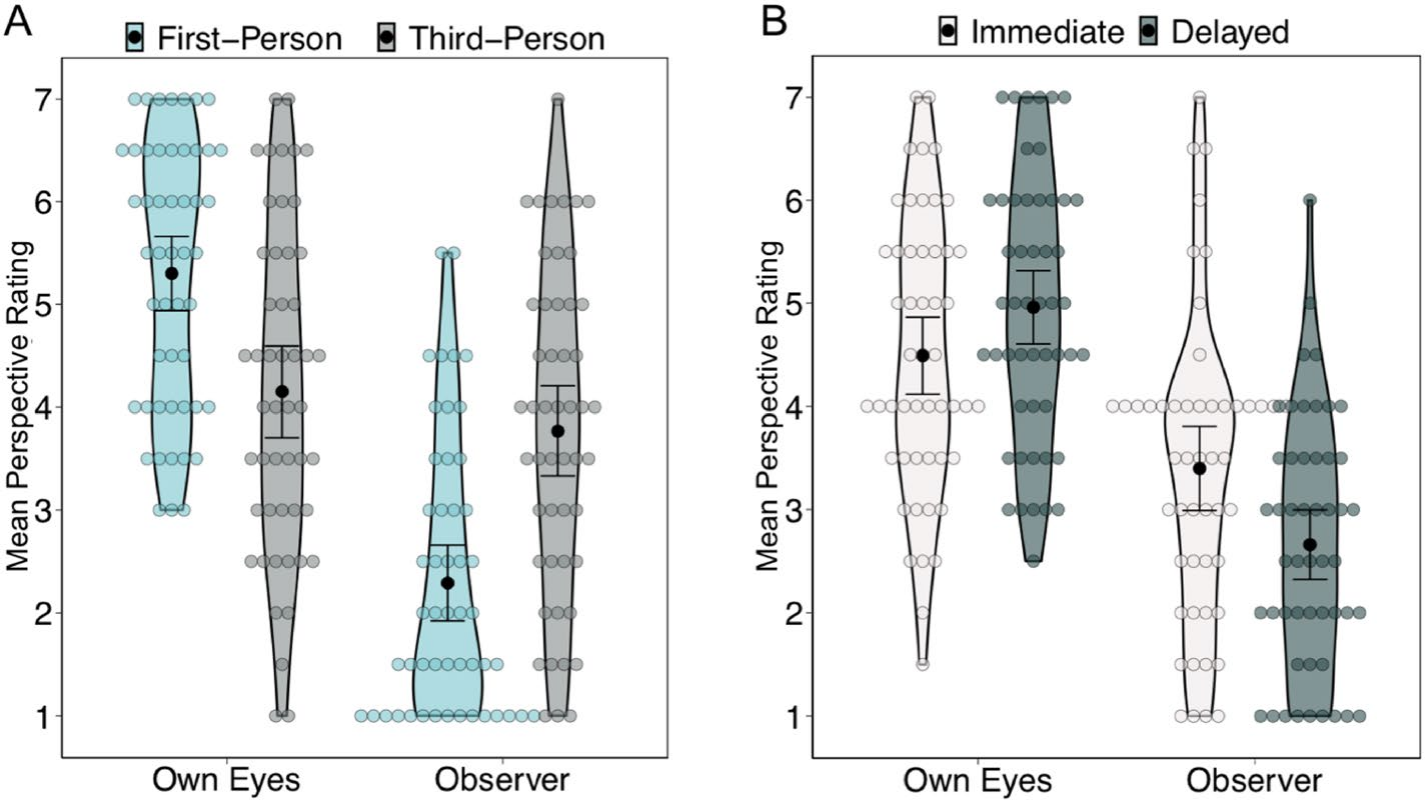
Visual perspective ratings. (**A**) Own eyes ratings were higher for memories of events experienced from a first-person than a third-person avatar perspective, whereas observer ratings were higher for third-person than first-person avatar perspectives. (**B**) Own eyes ratings were higher in memories retrieved following a delay, whereas observer ratings were lower following a delay. Colored circles reflect mean for each participant, black circles represent the mean within each condition, and error bars reflect the 95% CI.

There was a main effect of perspective rating, *F* (1,49) = 41.49, *p* < 0.001, $\eta_{p}^{2}$ = 0.46, reflecting higher ratings for own eyes (*M* = 4.73, *SD* = 1.09) than observer (*M* = 3.03, *SD* = 1.07) perspectives. There was also a main effect of perspective condition, *F* (1,49) = 4.12, *p* = 0.046, $\eta_{p}^{2}$ = 0.08, reflecting higher perspective ratings overall for 3PP (*M* = 3.96, *SD* = 0.65) compared to 1PP (*M* = 3.80, *SD* = 0.59) conditions. The main effects were qualified by a significant perspective rating x perspective condition interaction, *F* (1,49) = 24.55, *p* < 0.001, $\eta_{p}^{2} =$ 0.33 (see Fig. 5A). Bonferroni corrected post-hoc analyses revealed that own eyes ratings were higher for 1PP (*M* = 5.30, *SD* = 1.30) compared to 3PP (*M* = 4.15, *SD* = 1.61) conditions, but that observer ratings were higher for 3PP (*M* = 3.77, *SD* = 1.58) compared to 1PP (*M* = 2.29, *SD* = 1.33) conditions, both p’s < 0.001. There was also a perspective rating x time interaction, *F* (1,49) = 9.41, *p* = 0.004, $\eta_{p}^{2}$ = 0.16 (see Fig. 5B). Bonferroni corrected post-hoc analyses showed that own eyes perspective ratings increased across testing points (Immediate: *M* = 4.49, *SD* = 1.34; Delayed: *M* = 4.96, *SD* = 1.28, *p* = 0.03), whereas observer ratings decreased across testing points (Immediate: *M* = 3.40, *SD* = 1.47; Delayed: *M* = 2.66, *SD* = 1.22, *p* = 0.003). No other main effects or interactions were observed. Thus, these findings suggest that the visual perspective experienced during the formation of memories drives the visual perspective later adopted during memory retrieval.

We conducted additional 2(Perspective Condition: 1PP, 3PP) × 2(Test: Immediate, Delayed) repeated measures ANOVAs separately on average vividness, reliving, and emotional intensity ratings (for means and SD see Table 4). There was a main effect of test on vividness ratings, *F* (1,49) = 17.30, *p* < 0.001, $\eta_{p}^{2}$ = 0.26, reflecting higher ratings for memories tested immediately compared to a delay. There were no other main effects or interactions for the subjective ratings.

## Discussion: study two

The results of Study Two extend and complement the findings from Study One. We found that the use of personalized avatars led to a moderate degree of self-identification with the virtual avatar, consistent with the significant effects of avatar choice on the sense of presence found in Study One. We also found a significant increase in the sense of presence in virtual environments experienced from first-person compared to a third-person perspective. In contrast to Study One, in Study Two there were no significant differences in spatial memory accuracy in the first-person and third-person perspective conditions.

After controlling for differences in the FOV in Study Two, there were no significant differences in spatial memory accuracy in the first-person and third-person perspective conditions. Together, the findings across the two studies suggest that third-person perspectives may sometimes increase spatial memory accuracy by virtue of a wider FOV. While previous research has sometimes found that third-person perspectives lead to improved spatial awareness in virtual environments linked to increased FOV relative to first-person perspectives^14^ the present study is the first to directly test the relationship between visual perspective during encoding and spatial memory accuracy.

As in Study One, there were no significant effects of visual perspective on visual memory accuracy. Instead, visual details central to the task were recalled better than peripheral details, and there was also an overall reduction in memory accuracy following a delay. Consistent with this objective measurement of visual information, there were also no differences in subjective ratings of vividness in the first-person and third-person perspective conditions. Together these findings suggest that memories formed from first-person and third-person conditions contain a similar degree of visual information, in contrast with memories formed from first-person perspectives and later retrieved from a third-person perspective (i.e., a shift in visual perspective)^27^.

## General discussion

Across two studies, we investigated the influence of first-person and third-person avatar perspectives on memories for events experienced in immersive VR environments. Our findings provide greater understanding regarding the role of 1PP and 3PP experiences on memory formation. In both studies, we found no reliable differences in the accuracy of visual information. There were also no differences in subjective ratings of vividness, reliving, and emotional intensity memories for 1PP and 3PP experiences—even following a one-week delay as tested in Study Two. We did find that spatial memory accuracy for the layout of the scene was enhanced for events experienced from a 3PP relative to a 1PP in Study One, but not after controlling for the camera field of view in Study Two. Importantly, 3PP experiences did contribute to the formation of 3PP memories as reflected by a shift from 1PP to 3PP in subjective ratings during remembering, which provides empirical support for theories of visual perspective suggesting that memories can be formed from multiple visual perspectives^1,3^. Additionally, our findings provide evidence regarding the durability of alternative viewpoints overtime, with 1PPs dominating despite the origin of the perspective during encoding.

The subjective sense of presence was higher in immersive VR environments in which participants could choose their avatar (Study One) or for bespoke 1PP avatars that physically resembled the participant (Study Two). Experiencing a first-person avatar perspective enhances the feeling of becoming a virtual avatar, as opposed to controlling it from a third-person avatar perspective, which may in turn impact the sense of ownership. For example, Petkova, Khoshnevis, and Ehrsson^31^ performed a body swap illusion where participants wore an VR headset connected to a camera that provided either a 1PP or 3PP on a mannequin’s body. The authors then stroked both the participant’s actual body and the mannequin’s body in corresponding locations either synchronously or asynchronously. Synchronous visuo-tactile stimulation typically elicits a sense of illusory ownership over the mannequin’s body, whereas asynchronous visuo-tactile stimulation does not, due to the multisensory nature of neural systems underlying bodily selfhood^32,33^. Supporting the importance of visual perspective, transfer of bodily ownership, as measured by physiological reactions and questionnaire responses, was only evident when the mannequin was viewed from a 1PP. Similarly, a separate study conducted by Slater, Spanlang, Sanchez-Vives and Blanke^34^ found that adopting a 1PP was the strongest factor in establishing bodily ownership over a virtual avatar, compared to synchronous visuo-tactile stimulation and voluntary control over the avatar’s head movements. Collectively, this research suggests that 1PPs lead to a sense of bodily ownership, which allows one to project one’s own experiences to a body that is not one’s own, which may lead to a greater sense of presence within virtual environments. Thus, virtual environments may feel more real when experienced from a 1PP as it allows individuals to project their ownership, thoughts, and behavior onto their avatar^9^.

Third-person avatar perspectives might sometimes enhance spatial memory due to their wider camera FOV. In Study One we found more accurate spatial memory for 3PP than 1PP experiences, whereas in Study Two, there were no significant differences in spatial memory accuracy after controlling for differences in the camera FOV in 3PP and 1PP experiences. Our test of spatial memory required forming an allocentric or map-like representation of the virtual environment, which emphasized where objects were located with respect to other objects^35^. During memory retrieval, adopting a 3PP has been shown to increase recall for the spatial relationships between objects for mini-events encoded in the lab^22^. Some studies have suggested that 3PPs reflect an allocentric or viewpoint independent, rather than an egocentric or viewer centered perspective^36,37^. In contrast, Rubin and Umanath^38^ proposed that remembering always requires adopting a particular visual perspective, and have argued that both 1PPs and 3PPs are egocentric because they are centered with respect to where the representation of the physical self is located in the event. Whether or not 3PP experiences reflect allocentric or egocentric frames of reference, may depend upon the distance of the 3PP from the 1PP^39^. During memory retrieval, 3PPs can be located in one of several spatial locations with respect to the 1PP^40^, with a typical 3PP located at eye level, within 6 ft, and either in front or behind where the location of the 1PP in the memory. Thus, 3PPs may reflect both allocentric and egocentric frames of reference in memories, depending upon the location of the 3PP with respect to where the self (virtual or physical) is located. Future research should manipulate the location of third-person avatar perspectives in virtual environments and their impact on memory.

We found no differences in the amount of visual information retrieved in memories for 3PP and 1PP experiences, either when measured subjectively or objectively. In contrast, during memory retrieval 3PPs are associated with a reduction in vividness ratings^27^. One reason is that in these studies, people were asked to adopt a 3PP during retrieval of memories for 1PP experiences. Thus, changes in vividness reflect how actively shifting visual perspective influences visual information rather than differences in the amount of visual information encoded in memories for 3PP experiences. For example, Butler, Rice, Wooldridge, and Rubin^26^ found a reduction in subjective ratings of vividness when people were asked to repeatedly retrieve memories for 1PP experiences (both autobiographical memories and mini-events) from a 3PP across a one-month period. Reductions in the vividness of retrieval when shifting from 1PPs to 3PPs during rehearsal have also been shown to influence the accuracy of memories. Marcotti and St. Jacques^25^ found that differences in vividness ratings, when participants were instructed to adopt a 3PP versus a 1PP during memory retrieval for mini-events encoded in the lab, contributed to subsequent reductions in cued-recall accuracy for 3PP compared to 1PP conditions. Bergouignan and colleagues^17^ is the only study to our knowledge that has investigated visual information in memories for 3PP experiences. Across three retrieval repetitions, participants were allowed 24 s to retrieve memories for 3PP and 1PP experiences followed by a subjective vividness rating (exp. 3). They found a reduction in vividness ratings in memories for 3PP experiences, but only on initial retrieval attempts. In the current study, we instead gave participants unlimited time to retrieve memories rather than multiple repetitions, which may have contributed to the lack of significant differences in vividness ratings in memories for 3PP and 1PP experiences.

Our findings demonstrate that 3PP experiences can lead to the rapid formation of 3PP memories for recent events, as reflected by higher observer ratings and lower own eyes ratings during memory retrieval. Memories for recent experiences are typically associated with stronger own eyes than observer perspectives during remembering^29^. For example, St. Jacques, Szpunar, and Schacter^41^ asked people to recall over 200 autobiographical memories from the last five years, and then to provide phenomenological ratings including visual perspective. They found that 72% of memories were associated with a strong own eyes rating (i.e., >  = 5 own eyes ratings, <  = 3 observer ratings on 7-point scales from 1 = low to 7 = high). Other studies have suggested that the proportion of observer perspectives in recent memories is higher when taking into account the nature of the encoded event, with events involving a greater sense of self-consciousness (e.g., giving a public presentation) associated with stronger observer perspectives during remembering^40^. However, we also found that observer ratings were less durable overtime, as reflected by a greater reduction in observer ratings coupled with an increase in own eyes ratings following a one-week delay. Our 1PP is the default during remembering, with some people reporting that they rarely have 3PPs in memories^42^. Thus, for very recent memories, which retain the sense of vividness and other re-experiential aspects that support the ability to adopt a 1PP, people may tend to revert back to their dominant 1PP perspective following a short delay. Despite the effect of delay on perspective ratings, we did not find a significant effect of the retention interval on visual perspective ratings for 1PP and 3PP experiences, suggesting that people continued to recall 3PP experiences more strongly from a 3PP than 1PP even after a one-week delay. An important direction for future research will be to better understand how the visual perspective experienced during encoding contributes to visual perspective ratings during memory retrieval over more substantial delays, as well as considering individual differences that bias the durability of these effects overtime.

### Limitations

Our findings provide novel evidence about the nature of 3PP experiences in the formation of memory, but there are several limitations that will need to be addressed in future research. First, we tested a relatively small number of memories (e.g., 1 per condition), similar to previous investigations manipulating 3PP experiences using VR (e.g., 2 memories per condition in ^17^). Although our sample size was relatively large in both studies, the small number of trials might have reduced our ability to detect potential differences between the conditions.

Second, it is difficult to interpret overall differences between the two studies. In addition to camera FOV, the two studies differed in the nature of the avatar, the mirror-training task, the number and nature of the virtual environments, and how people could move their avatar within the environment (i.e., keypresses vs. body movement). Any of these methodological differences could have contributed to the differences we found in the sense of presence and spatial memory accuracy between the two studies. Future work should include these manipulations within the same study and individuals in order to better understand their influence.

A final limitation concerns the generalizability of findings in VR to reality. The extent to which the influence of 3PP experiences translates to real-world situations depends upon the extent to which participants treated their avatars as themselves within the virtual environment. Here we attempted to increase the sense of self-identification with the virtual avatar by allowing participants to choose their avatar (Study One) or by creating avatars that physically resembled the participants (Study Two), leading to an increase in the subjective sense of presence and moderately high ratings of self-identification, respectively. Ultimately, the artificial creation of 3PP experiences using VR may differ from the nature of 3PP experiences that occur in the “wild,” with resulting differences in memory for these experiences. However, the advantage of our approach is that it provides a paradigm to investigate these issues, while balancing ecological validity and experimental control.

### Conclusions

In the first empirical investigation of visual perspective in autobiographical memory, Nigro and Neisser (1983) suggested that “it is also possible to have observer experiences [i.e., 3PP experiences] . . . not all observer memories are produced by mnemonic distortion; some may accurately represent the original impression” (p. 468–69). The current study provides novel evidence supporting this idea using immersive VR by demonstrating that 3PP experiences create 3PP memories, as reflected by increased reports of adopting an observer viewpoint when remembering these recent events. Our results suggest that 3PP memories are as equally vivid and visually accurate as 1PP memories but might contain more accurate spatial information due to their naturally wider FOV. These findings may inform current theory regarding the nature 3PP in trauma memories where it is currently debated whether changes in visual perspective can occur during the formation of memories^43,44^. Novel therapies that capitalize on the ability of 3PPs to enhance spatial memory for lifelike VR environments could also be useful to retrain spatial awareness following stroke^45^. Given the increasing use of immersive VR to substitute reality, in future we may be exposed to an increasing number of 3PP experiences that will contribute to the formation of 3PP memories.

## Supplementary Information

Supplementary Information
