# Supplementary material for: Memories for third-person experiences in immersive virtual reality
Source: Sci Rep. 2021 Feb 25;11:4667. doi: 10.1038/s41598-021-84047-6 (PMC7907329; doi:10.1038/s41598-021-84047-6)
Supplement: Supplementary file 1 — Supplementary Information [file 41598_2021_84047_MOESM1_ESM.docx]

**Memories for Third-Person Experiences in Immersive Virtual Reality**

**Heather Iriye**^1+^ **and Peggy L. St. Jacques**^2*^

1. University of Sussex, School of Psychology, Brighton, UK

2. University of Alberta, Department of Psychology, Edmonton, Canada

*peggy.stjacques@ualberta.ca

+ Present address: Karolinska Institutet, Department of Neuroscience, Solna, Sweden

# Supplementary Methods: Narrative Recall

Narrative accounts were analyzed using the Linguistic Inquiry and Word Count (LIWC)^1^, a text-analysis tool that assesses the frequency with which various categories of words are used. Here, we analyzed the number of words related to perceptual processes (e.g., feels, touch), affect (i.e., words relating to positive emotion and negative emotions of anxiety, anger, and sadness), and first-person (i.e., I, we) and third-person pronouns (i.e., she/he, they).

# Supplementary Results: Narrative Recall

**Study One.** There were no differences in the overall number of words used to describe memories experienced from first-person or third-person perspectives. Separate 2 (Perspective Condition: 1PP, 3PP) x 2 (Avatar Group: Choice, No Choice) mixed repeated measures ANOVAs with perspective condition as a within-subjects factor and avatar group as a between-subjects factor were conducted on the proportion of words related to perceptual processes, affect, and first-person and third-person pronouns from the LIWC analysis (for means and SD see Supplementary Table).

Turning to words related perceptual processes related to feeling, there was a significant main effect of perspective, *F* (1, 47) = 4.12, *p* = .048, $\eta_{p}^{2}$ = .081. Narratives in the 1PP condition contained more words pertaining to feeling states (*M* = 2.07, *SD* = 3.27), compared to the 3PP condition (*M* = 1.10, *SD* = 1.79). There were no other main effects or interactions. Upon examining the individual memory narratives, however, we noticed that several of the narratives exclusively in the 1PP condition referred to dizziness and/or nausea due to VR sickness (*N* = 6). When we excluded these details from the narrative analysis the main effect of perspective condition did not remain, suggesting that the differences in words related to feeling states was due to the increase in VR sickness experienced in the 1PP compared to the 3PP conditions.

**Study Two.** There were no differences in the overall number of words used to describe memories in the first-person or third-person perspective conditions. Separate 2 (Perspective Condition: 1PP, 3PP) x 2 (Test: Immediate, Delay) mixed repeated measures ANOVAs with perspective condition as a within-subjects factor and avatar group as a between-subjects factor were conducted on the proportion of words related to perceptual processes, affect, and first-person and third-person pronouns from the LIWC analysis (for means and SD see Supplementary Table). Turning to average perceptual processes, there was a main effect of test, *F* (1,48) = 8.72, *p* = .005, $\eta_{p}^{2}$ = .15, reflecting a greater proportion of words related to perceptual processes overall to describe narratives when memory was tested immediately (*M* = 5.13, *SD* = 2.79) compared to after a delay (*M* = 3.93, *SD* = 1.91). There were no other main effects or interactions.

# Supplementary Discussion

One limitation of Study One was that participants also experienced a greater sense of VR sickness in the 1PP than 3PP conditions, which may have inadvertently distracted participants from focusing on the main experimental task. In Study Two, we minimized the degree of VR sickness by using motion capture software that allowed participants to interact more naturally within the virtual environments (e.g., number of participants reporting VR sickness: Study One = 14, Study Two = 3). As expected, minimizing VR sickness attenuated differences in the amount of perceptual words related to feelings in narratives in the first-person and third-person perspective conditions, suggesting that differences in feeling words in Study One were due to the increase in VR sickness in the first-person perspective condition rather than a greater focus on perceptual aspects of memory.

1. Pennebaker, J., Francis, M., & Booth, R. (2001). Linguistic Inquiry and Word Count (LIWC): LIWC2001. *71*.

| **Supplementary Table.** *Narrative Recall* | | |  |  |  |  |  |  |
| --- | --- | --- | --- | --- | --- | --- | --- | --- |
|  | **Study 1** | | | | **Study 2** | | | |
|  | Avatar Choice | | No Avatar Choice | | Immediate | | Delayed | |
|  | Mean | SD | Mean | SD | Mean | SD | Mean | SD |
| *Total Word Count* |  |  |  |  |  |  |  |  |
| First-Person | 60.88 | 45.89 | 57.04 | 30.76 | 81.27 | 34.64 | 76.39 | 35.29 |
| Third-Person | 55.68 | 30.57 | 64.58 | 31.78 | 80.10 | 34.96 | 72.94 | 32.39 |
| *Perceptual Words* |  |  |  |  |  |  |  |  |
| First-Person | 4.60 | 3.32 | 6.22 | 4.27 | 5.50 | 3.45 | 3.96 | 2.45 |
| Third-Person | 4.22 | 3.77 | 5.25 | 4.73 | 4.77 | 3.53 | 3.91 | 2.63 |
| *Feeling Words* |  |  |  |  |  |  |  |  |
| First-Person | 2.00 | 3.28 | 2.14 | 3.26 | 0.64 | 0.96 | 0.36 | 0.75 |
| Third-Person | 0.78 | 1.60 | 1.41 | 1.97 | 0.53 | 0.92 | 0.40 | 0.82 |
| *Affect Words* |  |  |  |  |  |  |  |  |
| First-Person | 3.47 | 3.65 | 2.73 | 2.97 | 0.79 | 1.11 | 0.77 | 1.42 |
| Third-Person | 4.32 | 4.10 | 3.25 | 3.79 | 1.34 | 2.05 | 0.62 | 0.96 |
| *First-Person Pronouns* | |  |  |  |  |  |  |  |
| First-Person | 4.57 | 4.04 | 3.31 | 3.53 | 4.01 | 2.87 | 3.86 | 3.16 |
| Third-Person | 4.62 | 3.60 | 3.72 | 3.66 | 4.61 | 3.48 | 3.68 | 3.24 |
| *Third-Person Pronouns* | |  |  |  |  |  |  |  |
| First-Person | 0.03 | 0.13 | 0.16 | 0.60 | 0.18 | 0.43 | 0.27 | 0.62 |
| Third-Person | 0.28 | 0.71 | 0.27 | 0.64 | 0.28 | 0.57 | 0.31 | 1.00 |
|  |  |  |  |  |  |  |  |  |
